# Subclinical Leptospiral Infections and Antibody Waning During Pregnancy: A Population-Based Cohort Study from Rural Sri Lanka

**DOI:** 10.4269/ajtmh.25-0553

**Published:** 2026-03-19

**Authors:** Suneth Agampodi, Dinesha Jayasundara, Janith Warnasekara, Thilini Agampodi, Indika Senavirathna, Dilrukshi Menike, Digantha Aswaddumage, Madushika Sewwandi, Chamila Kappagoda, Anuruddhi Premalal, Hwa Young Kim, Shashanka Rajapakse, Raphaël M. Zellweger

**Affiliations:** ^1^ Innovation, Initiatives & Enterprise Development, International Vaccine Institute, Seoul, Republic of Korea;; ^2^ Department of Community Medicine, Faculty of Medicine and Allied Sciences, Rajarata University of Sri Lanka, Saliyapura, Sri Lanka;; ^3^ Department of Microbiology, Faculty of Medicine and Allied Sciences, Rajarata University of Sri Lanka, Saliyapura, Sri Lanka;; ^4^ Epidemiology, Public Health, Impact Unit, International Vaccine Institute, Seoul, Republic of Korea;; ^5^ Department of Biochemistry, Faculty of Medicine and Allied Sciences, Rajarata University of Sri Lanka, Saliyapura, Sri Lanka;; ^6^ Department of Physiology, Faculty of Medicine and Allied Sciences, Rajarata University of Sri Lanka, Saliyapura, Sri Lanka;; ^7^ Department of Public Health & AI, Graduate School of Cancer Science and Policy, National Cancer Center, Goyang-si, Republic of Korea

## Abstract

Leptospirosis is a zoonotic infection with significant mortality, yet its burden is underestimated due to diagnostic limitations. The dynamics of naturally acquired anti-*Leptospira* antibodies, particularly during pregnancy, remain poorly understood. This study examined the seroincidence of leptospiral infection among a cohort of pregnant women and characterized longitudinal antibody kinetics in an endemic setting. Paired sera were collected from a population-based pregnancy cohort in Anuradhapura, Sri Lanka. Women were enrolled before 12 weeks of gestation and followed up between 25–28 weeks. Data on sociodemographics, self-reported febrile illness, and hospital admissions were collected. Microscopic agglutination testing (MAT) was performed against 11 *Leptospira* serovars. Seroincidence was defined as seroconversion from non-reactive (<1:50) to reactive (≥1:50). Antibody kinetics were assessed using Gaussian accelerated failure time and proportional odds logistic regression models. Among 3,374 recruited women, 1,338 had paired samples. Of 1,245 seronegative at baseline, 22 (1.8%) seroconverted, yielding a seroincidence rate of 52.8/1,000 person-years (95% CI: 31.2–76.8), using the ≥1:50 definition; using a ≥1:100 cutoff, the seroincidence rate was 14.4 per 1,000 person-years (95% CI: 5.3–31.4). No significant differences in pregnancy outcomes were observed between seroconverts and non-seroconverts. Among 93 women with baseline antibody titers ≥1:50, 14 (15.1%) seroreverted to <1:50, 12 (12.9%) showed a decline in titers but remained ≥1:50, 56 (60.2%) remained unchanged, and 11 (11.8%) showed an increase in antibody titers. Antibody decay rates ranged from +0.353 to –0.789 log_2_ units/30 days (mean: –0.074), with faster decay at higher baseline titers. This study reveals frequent, asymptomatic *Leptospira* exposure and rapid antibody decline during pregnancy, highlighting substantial silent transmission and the limitations of current surveillance tools in endemic settings.

## INTRODUCTION

Leptospirosis is a globally widespread but neglected zoonotic disease, endemic in more than 150 countries, with more than 1 billion people at risk of infection. Despite this wide distribution, it remains underdiagnosed and underreported due to its broad clinical spectrum and nonspecific presentation, which overlap with other tropical febrile illnesses such as dengue and malaria.^1^^–^^3^ Clinical suspicion is often low, and the lack of reliable point-of-care diagnostic tools further hinders the timely detection and management of these conditions. Although the pathogen is susceptible to first-line antibiotics, inadequate diagnostic capacity contributes to delayed treatment, severe complications, and preventable mortality.

The microscopic agglutination test (MAT), considered the reference standard for serological diagnosis, detects antibodies only after the first week of illness and requires live bacteria as an antigen, significant technical expertise, and access to reference laboratories.^1^^,^^4^^–^^7^ Its sensitivity is limited in the acute phase and may miss up to 80% of early infections.^1^ Although several commercial assays report acceptable diagnostic accuracy, most have been validated against MAT, which itself is suboptimal in early disease, leading to underestimated sensitivity in clinical settings.^8^^–^^14^ Molecular diagnostics, such as quantitative polymerase chain reaction (qPCR), offer greater accuracy but are not widely implemented in endemic settings and still face challenges related to assay standardization and field-level applicability.^15^^–^^18^

Because of the limitations of available diagnostics, treating physicians frequently rely on clinical judgment to diagnose leptospirosis. Icteric leptospirosis, or Weil’s disease, represents a recognizable severe form but occurs in only 5–15% of symptomatic cases.^19^ Most infections present as the anicteric form with nonspecific symptoms, making clinical diagnosis highly unreliable and often resulting in misclassification as other febrile illnesses. Consequently, a substantial proportion of leptospiral infections remain undiagnosed and unreported, particularly in low- and middle-income countries (LMICs) with under-resourced health systems.

This diagnostic uncertainty contributes to significant discrepancies in global burden estimates. Clinical surveillance data often fail to capture the true incidence, particularly in settings where widespread asymptomatic or subclinical infections are prevalent. Accurate burden estimation requires prospective population-based cohort studies that use paired serological testing to capture both symptomatic and asymptomatic infections. However, such studies remain rare, with most available data limited to specific high-risk settings such as urban slums in Brazil.^20^^–^^23^

Furthermore, little is known about the serological kinetics of anti-*Leptospira* antibodies following natural exposure. Studies from Brazil have shown that antibody titers measured by MAT can decline over relatively short periods, particularly among asymptomatic or mildly infected individuals,^21^^,^^22^ suggesting that waning immunity may lead to underestimation of true seroincidence in longitudinal surveys. These findings raise important questions regarding the use of MAT-based seroprevalence for estimating population immunity or exposure history.

Pregnancy presents an important and understudied context for leptospirosis surveillance. Immunological changes during pregnancy may influence the course of infection and antibody kinetics. A recent systematic review indicates that the incidence of leptospirosis among pregnant women presenting with fever is 1.3 per 10,000.^24^ There are no published cohort studies assessing the seroincidence of leptospirosis among healthy pregnant women. Given the potential risks of infection during pregnancy—including febrile illness, adverse pregnancy outcomes, and unrecognized fetal exposure—there is a critical need for cohort-based evidence on leptospiral infection in pregnant women.^24^

To address these knowledge gaps, we conducted a serological substudy within the Rajarata Pregnancy Cohort (RaPCo) in rural Sri Lanka. Our objectives were 2-fold: 1) to estimate the seroincidence of leptospiral infection during pregnancy using paired MAT data, and 2) to characterize antibody kinetics over time, including patterns of seroconversion, seroreversion, and titer decay.

## MATERIALS AND METHODS

### Study design and participants.

This study was conducted within the RaPCo, a large-scale, population-based, prospective maternal cohort established in the Anuradhapura District, Sri Lanka. Between July and September 2019, pregnant women were enrolled at the time of registration with the public health system (<12 weeks’ gestation), and the baseline assessment was conducted at community-based RaPCo clinics, replacing routine first-trimester visits. This process achieved 89.8% coverage of eligible pregnant women in the district during the study period.^25^ All participants underwent a comprehensive baseline assessment and were followed up during the late second or early third trimester of pregnancy. For the present analysis, we included all women who completed follow-up and had paired serum samples available for serological testing.

### Sample collection and laboratory procedures.

Venous blood was collected at baseline and follow-up.^6^ The MAT antigen panel comprised 11 live *Leptospira* serovars, including globally representative strains—*L. interrogans* (serovars Bratislava, Icterohaemorrhagiae, Canicola, Mankarso, Bataviae, and Wolfii), *L. sanntarosai* (Georgia and Pyogenes), and two locally isolated clinical strains (*L. interrogans* serovar Weerasinghe, and *L. borgpetersenii* serovar Ceylonica). In addition, the panel included the saprophytic strain *L. biflexa* serovar Patoc. Each serum sample was initially screened at a 1:50 dilution; reactive samples were further titrated in 2-fold serial dilutions up to 1:3,200. MAT titers were defined as the highest serum dilution demonstrating ≥50% agglutination of motile *Leptospira* under dark-field microscopy. The highest titer recorded across the 11-serovar panel was used as the maximum titer for each participant at each time point.

### Seroincidence estimation.

Seroincidence was defined as seroconversion, i.e., a change from nonreactive (<1:50) to reactive (≥1:50) against any *Leptospira* serovar between the baseline and follow-up assessments. Participants with reactive titers at baseline were excluded from the incidence cohort. A threshold of ≥1:50 was used to define seropositivity. The exact time interval between baseline and follow-up sampling was calculated in days and converted to person-years for incidence rate estimation. Incidence was reported as the number of seroconversions per 1,000 person-years, with 95% confidence intervals derived using the Poisson exact method.

A sensitivity analysis was conducted using a more stringent seroconversion definition, requiring a follow-up titer of ≥1:100 to be classified as reactive. Serovar-specific reactivity was recorded and analyzed descriptively, including the distribution of maximum titers and the number of serovars agglutinated per participant.

### Clinical outcomes and exploratory analysis.

Self-reported febrile illness during the follow-up period was captured through structured interviews at the follow-up visit and supplemented with data from a district-wide hospital admission surveillance system. Pregnancy outcomes—including gestational age at delivery and neonatal birth weight—were extracted from institutional records and verified or supplemented via follow-up telephone interviews. Preterm birth was defined as delivery before 37 completed weeks of gestation, and low birth weight as <2,500 g.

Exploratory analyses compared clinical outcomes between seroconverters and non-seroconverters using χ^2^ and *t*-tests as appropriate. Logistic regression models were constructed to explore potential predictors of seroconversion.

### Antibody kinetics and statistical analysis.

To evaluate anti-*Leptospira* antibody kinetics during pregnancy, we used three complementary approaches. First, participant-level decay rates were estimated as the change in log_2_-transformed maximum MAT titer per 30 days and regressed on baseline titer. Second, a Gaussian accelerated failure time (AFT) model was fitted using log_2_-transformed follow-up titers as the outcome and follow-up duration as the predictor, accounting for left-censoring below 1:50 (coded as log_2_[25]). Third, MAT titers were categorized into ordinal groups (<1:50, 1:50–1:100, 1:200–1:400, ≥ 1:800), and a proportional odds logistic regression model was used to assess temporal trends. In both models, a secondary analysis excluded participants with rising titers. All analyses were performed using R (v. 4.5.0; R Development Core Team); figures were generated with ggplot2 (v. 3.4.0) and refined externally.

## RESULTS

### Study sample.

A total of 3,374 pregnant women were enrolled in the RaPCo cohort at baseline. Of these, 1,450 completed at least one scheduled follow-up visit, and paired serum samples were available from 1,338 participants who formed the subset for serological analyses.

**Figure 1.**
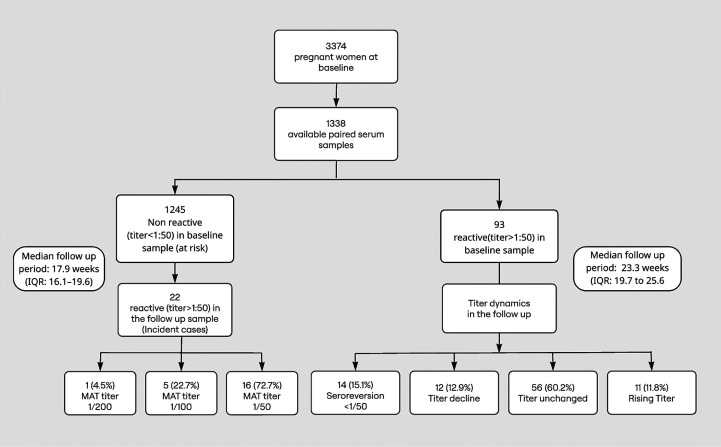
Serological reactivity for leptospirosis among Rajarata Pregnancy Cohort pregnant women.

Of those, 1,245 pregnant women who had no reactivity (titer <1:50) in the baseline sample were included in the seroincidence cohort (Figure 1). The median age of the cohort was 28 years (interquartile range [IQR], 24–32 years), and the median gestational age at enrollment was 9.0 weeks (IQR: 7.0–10.0 weeks). Most participants were of Sinhala ethnicity (88.8%), followed by Sri Lankan Moors (9.2%), Malays (1.0%), and Tamils (0.9%) in origin. A prior history of leptospirosis was self-reported by only four individuals (0.4%), and no clinical or laboratory confirmation was available. Table 1 summarizes the baseline demographic and obstetric characteristics of the study population.

**Table 1:** Baseline characteristics of pregnant women included in the seroincidence analysis (*n* = 1,245)

Characteristic	*n*	%
Maternal age group (years)		
<20	109	8.8
20–29	675	54.2
30–39	440	35.3
≥40	21	1.7
Ethnicity		
Sinhala	1,106	88.8
Moor	115	9.2
Tamil	11	0.9
Malay	13	1.0
Maternal education		
Not completed primary	18	1.5
Completed primary	101	8.2
Post primary	660	53.4
Secondary	458	37.0
Gravidity		
1	386	31.0
2	387	31.1
3	314	25.2
4	120	9.6
≥5	37	3.0
Self-reported leptospirosis history		
Yes	4	0.4
No	1,120	99.6

### Seroincidence of leptospiral infection.

**Figure 2.**
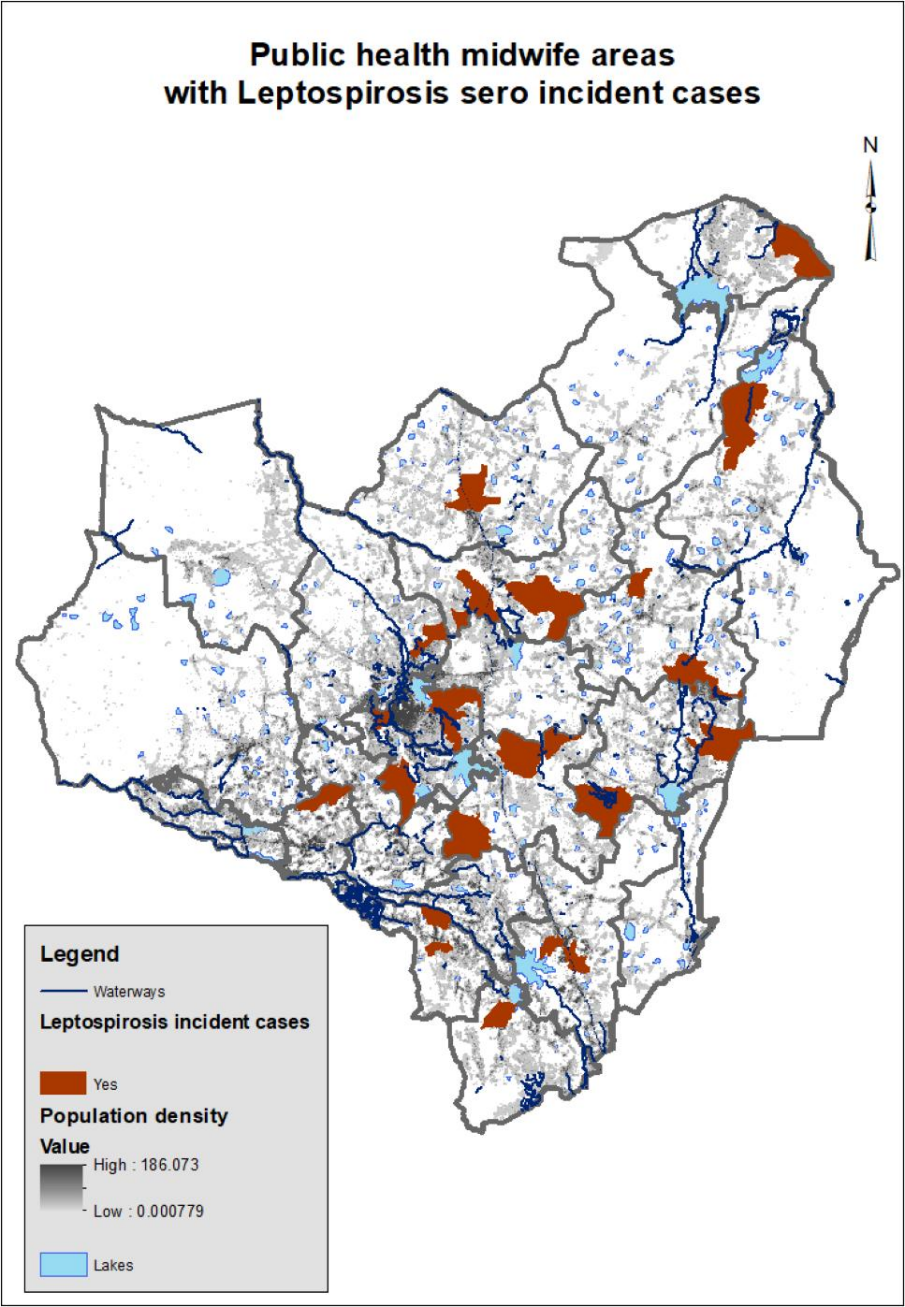
Distribution of PHM areas with leptospirosis seroincident cases in Anuradhapura district.

The follow-up period contributed to a total of 416.9 person-years of observation with a median of 17.9 weeks (IQR: 16.1–19.6) between baseline and follow-up sampling. The median gestational age at follow-up was 27.0 weeks (IQR: 25.6–28.3 weeks). Twenty-two women (1.8%) developed serological evidence of leptospiral infection during pregnancy, corresponding to a seroincidence rate of 52.8 per 1,000 person-years (95% CI: 31.2–76.8) (Figure 1). Using a more stringent MAT cutoff (≥1:100, *n* = 6), the seroincidence was 14.4 per 1,000 person-years (95% CI: 5.3–31.4). Leptospirosis incident cases were reported from 22/307 Public Health Midwife (PHM) areas (Figure 2).

### Serological reactivity patterns.

Among seroconverters, the highest MAT titer observed at follow-up was 1:200 in 1 (4.5%), 1:100 in 5 (22.7%), and 1:50 in 16 participants (72.7%). No participant exhibited a titer ≥1:400, the conventional diagnostic threshold for acute clinical leptospirosis (Figure 1).

Analysis of serovar-specific agglutination patterns revealed that 20 of 22 seroconverters exhibited agglutination with *L. interrogans* serovar Bratislava at titers ≥1:50 (Table 2). In 19 of these cases, Bratislava yielded the highest observed titer. Agglutination with serovar Icterohaemorrhagiae was detected in three participants, including one whose serum exhibited no reactivity to any other serovar. Dual agglutination involving serovar Weerasinghe was observed in two participants, and one participant had isolated reactivity to serovar Pyrogenes. Overall, three participants (13.6%) showed agglutination with more than one serovar.

**Table 2:** Highest MAT titers with selected *Leptospira* serovars among seroconverters (*n* = 22)

No. of Participants	Serovar Bratislava	Serovar Icterohaemorrhagiae	Serovar Weerasinghe	Serovar Pyrogenes	Comment
1	1:200	–	–	–	Single-serovar: Bratislava
3	1:100	–	–	–	Single-serovar: Bratislava
1	1:100	–	1:50	–	Dual-serovar: Bratislava + Weerasinghe
1	1:50	1:100	1:50	–	Triple-serovar: Icterohaemorrhagiae + Bratislava + Weerasinghe
1	1:50	1:50	–	–	Dual-serovar: Bratislava + Icterohaemorrhagiae
13	1:50	–	–	–	Single-serovar: Bratislava
1	–	–	–	1:50	Single-serovar: Pyrogenes
1	–	1:50	–	–	Single-serovar: Icterohaemorrhagiae

– (dashes) = no agglutination at titers ≥1:50; MAT = microscopic agglutination testing.

An exploratory logistic regression was conducted to assess potential predictors of seroconversion, including residence, ethnicity, maternal age at conception, gestational age at enrollment, and trimester at follow-up. Due to the small sample size (*n* = 22), model convergence was limited, and adjusted estimates are not reported.

### Clinical disease and pregnancy outcomes.

Febrile illness during the follow-up period was reported by 3 of the 22 seroconverters (13.6%). Among non-seroconverters, 18.3% (*n* = 219) reported febrile illness during the follow-up. None of the affected individuals were hospitalized or received a clinical diagnosis of leptospirosis.

All pregnancies among seroconverters resulted in live births, and no stillbirths were reported. The mean birth weight among seroconverters was 2,896.6 g, compared with 2,907.1 g in the seronegative group (*P* = 0.92). The gestational age at delivery was 38.3 weeks in seroconverters and 38.5 weeks among seronegative women (*P* = 0.52). No statistically significant differences were observed in pregnancy outcomes between seroconverters and non-seroconverters.

### Antibody kinetics among baseline seropositive participants.

Of the total cohort, 93 pregnant women had baseline titers of ≥1:50 and follow-up samples available. Among those, with paired MAT results, 14 individuals (15.1%) seroreverted to titers <1:50 at follow-up. A further 12 participants (12.9%) experienced a decline in titers. Stable titers were observed in 56 participants (60.2%), and 11 (11.8%) exhibited an increase in titer over time. Among those with increasing titers, all showed a 2-fold rise—from 1:50 to 1:100 (*n* = 7), from 1:100 to 1:200 (*n* = 3), or from 1:200 to 1:400 (*n* = 1). None demonstrated a 4-fold or greater increase (Figure 1).

Among participants with declining but still reactive titers, 11 of 12 (91.7%) had a 2-fold decrease, and 1 participant had a 4-fold decrease (Table 3). The stratified analysis revealed a titer-dependent pattern in the frequency of titer decline and seroreversion. Among participants with a baseline titer of 1:200 (*n* = 24), 13 (54.2%) experienced a decline in titer, including 6 (25.0%) who seroreverted. In the 1:100 group (*n* = 32), 9 participants (28.1%) declined, and 4 (12.5%) seroreverted. Among those with a baseline titer of 1:50 (*n* = 35), 3 participants (8.6%) seroconverted. Of the 2 participants with higher baseline titers, the one with a 1:400 titer seroreverted, whereas the one with a 1:800 titer retained the exact titer at follow-up.

**Table 3:** Participant-level change in maximum MAT titer and magnitude of decline in seropositive at baseline (*n* = 93)

Titer Change Category	*n*	%	Fold Decrease
Seroreversion (<1:50 at follow-up)	14	15.1	Not quantified
Decline (titer ≥1:50 at follow-up)	12	12.9	2-fold (*n* = 11); 4-fold (*n* = 1)
Stable titer	56	60.2	–
Increased titer	11	11.8	2-fold (*n* = 11)
Total	93	100.0	–

– (dash) = not relevant; MAT = microscopic agglutination testing.

**Figure 3.**
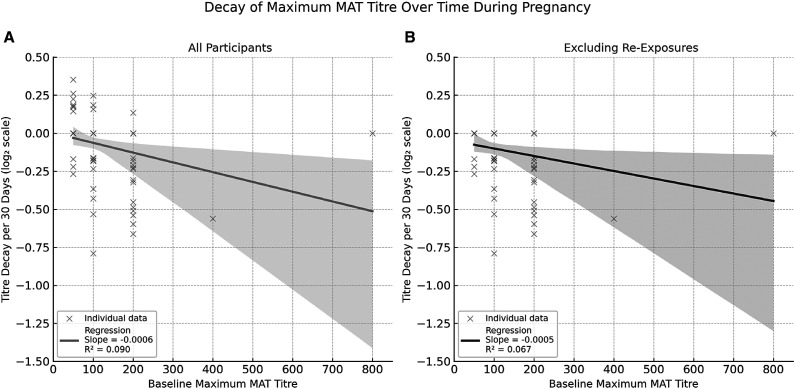
Decay of maximum microscopic agglutination testing titer over time stratified by baseline titer group.

To quantify antibody waning during pregnancy, individual-level decay rates were calculated using paired samples from 93 participants with baseline MAT titers ≥1:50. Decay was defined as the change in log_2_-transformed maximum titer per 30 days. Among all participants, decay rates ranged from +0.353 to –0.789 log_2_ units per 30 days, with a mean of –0.074. Linear regression of decay rates on baseline titers yielded a slope of –0.00064 log_2_ units per 30 days per unit increase in titer (intercept = 0.0016; *R*^2^ = 0.090) (Figure 3A)—a secondary analysis excluded 11 participants whose titers increased at follow-up, suggestive of reexposure or boosting. Among the remaining 82 participants, the mean decay rate was –0.111 log_2_ units per 30 days (range: 0.0 to –0.789). In this restricted cohort, the regression model yielded a slope of –0.00049 (intercept = –0.0507; *R*^2^ = 0.067) (Figure 3B).

To evaluate titer changes over time using model-based approaches, a Gaussian AFT model was fitted with log_2_-transformed follow-up titers as the outcome and follow-up duration as the predictor, accounting for left-censoring at titers <1:50 (coded as log_2_[25]). In the full cohort (*n* = 93), the AFT model yielded a nonsignificant positive slope (β = 0.0044, SE = 0.0030, *P* = 0.15). When restricted to participants without reexposure (*n* = 82), the slope remained nonsignificant (β = 0.0034, SE = 0.0034, *P* = 0.31). To complement this, follow-up titers were categorized into ordinal levels (<1:50, 1:50–1:100, 1:200–1:400, ≥1:800), and proportional odds logistic regression was performed using follow-up duration as the predictor. The model yielded nonsignificant trends in both the full cohort (β = 0.0074, SE = 0.0071, *P* = 0.30) and the restricted cohort (β = 0.0057, SE = 0.0080, *P* = 0.48), consistent with stable or only mildly waning antibody titers during pregnancy.

## DISCUSSION

This study provides the first population-based estimate of *Leptospira* seroincidence during pregnancy in an endemic setting. The high frequency of asymptomatic seroconversion, coupled with low peak antibody titers, suggests widespread subclinical transmission that remains undetected by routine surveillance. Although modeling of antibody decay yielded no statistically significant association between follow-up time and titer, individual-level estimates suggested a modest and variable decline, particularly among those with higher initial titers. These trends should be interpreted cautiously given the ordinal nature of MAT data and the small number of seroconversions, which limited model precision. These findings reinforce the transient nature of MAT-detectable responses in subclinical infections and underscore the need to interpret serological data within the limitations of assay resolution and host immune modulation during pregnancy.

The estimated seroincidence rate of 52.8 per 1,000 person-years (MAT ≥1:50) is consistent with findings from prospective studies in high-risk settings in Brazil, Colombia, and Peru (range: 35–130 per 1,000),^21^^–^^23^^,^^26^^–^^28^ although those studies applied varying seroconversion definitions and MAT thresholds (e.g., ≥1:25 or ≥1:100), which limits direct comparability. It is, however, lower than estimates from India.^29^ Similar rates have been reported in high-risk populations within low-endemicity countries such as the United States, Spain, and New Zealand.^30^^–^^32^ In Vietnam, a lower incidence was reported among children (22 per 1,000 person-years),^33^ likely reflecting differences in exposure patterns and immune response kinetics between pediatric and adult populations. Although epidemiological risk factors differ from those in pregnant women, both groups represent physiologically distinct populations in whom antibody dynamics and clinical recognition of leptospirosis remain understudied. In our cohort, participants were drawn from the general rural population without any known exposure to outbreaks, suggesting that endemic transmission is sustained through routine agricultural and peri-domestic activities. When a more stringent seroconversion threshold (MAT ≥1:100) was applied, the incidence declined to 14.4 per 1,000 person-years, underscoring the impact of surveillance definitions on burden estimates.

Only 13.6% of seroconverters reported febrile illness, and none were clinically diagnosed with leptospirosis, consistent with prior studies showing most infections are subclinical.^23^^,^^30^^,^^34^ This finding has specific relevance in pregnancy, where leptospirosis is underdiagnosed^35^ and can mimic obstetric conditions such as preeclampsia, HELLP (Hemolysis, Elevated Liver enzymes and Low Platelets) syndrome, and acute fatty liver.^36^ An important consideration is that leptospirosis is also a recognized cause of maternal deaths in Sri Lanka, showing the critical need for earlier detection and targeted surveillance in this population. From 2006 to 2021, seven maternal deaths were directly attributed to leptospirosis in Sri Lanka, according to official maternal death reviews.^37^^–^^40^

This silent transmission contributes to substantial under-ascertainment in clinical surveillance systems. Costa et al. estimated an annual incidence of clinical disease as 3 per 1,000 cases in Sri Lanka,^41^ more than 30-fold lower than our seroincidence estimate. This disparity highlights underdetection, particularly among populations not routinely prioritized in surveillance, such as pregnant women engaged in agriculture. Clinical studies have reported fetal loss rates exceeding 50% in confirmed maternal cases,^42^ yet high-quality epidemiological data on leptospirosis in pregnancy remain scarce.^24^

The antibody response profile among seroconverters was characterized by low peak titers, with 72.7% reaching only 1:50 and none exceeding 1:400. This pattern suggests a low-inoculum exposure to pathogenic *Leptospira* or exposure to nonpathogenic *Leptospira* species, resulting in transient IgM responses without progression to long-lasting, class-switched immunity. Pregnancy-associated immunomodulation, including a Th2-biased immune environment, may further suppress durable antibody production. The dominant reactivity to *L. interrogans* serovar Bratislava is consistent with earlier Sri Lankan studies involving the dry zone and showing frequent responses to Australis-group serovars in both clinical and subclinical infections.^43^^,^^44^ Given the presence of free-roaming livestock in the region, zoonotic transmission from cattle or buffalo remains a plausible reservoir source. Although cross-reactivity cannot be excluded, the consistent pattern of Bratislava dominance warrants further investigation into its epidemiological significance and inclusion in local surveillance panels.

Antibody decay in our cohort was modestly titer-dependent, with faster declines observed among individuals with higher initial titers (1:200–1:400). Unlike clinical cohorts where MAT titers ≥1:800 can persist for years,^45^ our findings in a naturally exposed community setting align with studies from Brazil showing that low-to-moderate titers often wane within 3 months.^21^ These rapid declines likely reflect lower antigenic stimulation and the transient nature of subclinical infections. Our results reinforce known limitations of MAT, including discrete dilution steps and isotype insensitivity, which can obscure decay dynamics and bias incidence estimates.^22^ The absence of significant associations in model-based analyses supports the interpretation that MAT may underdetect waning in subclinical or low-exposure infections, consistent with its known limitations in resolution and sensitivity. As shown in modeling studies, accounting for waning is essential to avoid underestimating seroincidence.^22^^,^^46^ Public health surveillance in endemic settings should consider more frequent sampling or decay-adjusted models to capture transient immune responses and improve burden estimates. A subset of participants (11.8%) showed rising titers over time, indicating possible boosting from reexposure. Similar findings have been reported elsewhere,^21^ complicating the interpretation of serology in the absence of molecular diagnostics or exposure histories. During pregnancy, immune responses may be further altered, making it challenging to distinguish between new infections and secondary immune responses.

These findings have several key implications. First, the high rate of asymptomatic seroconversion, coupled with poor clinical recognition and reliance on insensitive diagnostics, indicates that silent transmission is widespread. Second, the frequent occurrence of subclinical infection during pregnancy, combined with evidence of fetal risks from prior studies, shows the need to include pregnant women in leptospirosis surveillance and risk assessments explicitly. Although no adverse outcomes were observed in this cohort, this frequent occurrence of subclinical infection may reflect either actual absence or underpowered detection. Third, the observed titer-dependent antibody decay and serological variability highlight the limitations of MAT in estimating exposure and immunity. These findings support the development and application of high-resolution, isotype-specific assays to improve surveillance accuracy, define correlates of protection, and guide maternal vaccine development efforts.

### Limitations.

Although these data provide important insights, generalizability remains limited. Immunological features of pregnancy, such as altered B-cell responses, may not reflect decay or response profiles in other populations. Rural exposure patterns also differ from those in urban slum environments, where most prior data originate. MAT, despite its reference status, is semiquantitative and lacks isotype resolution. Its 2-fold dilution structure and operator variability limit kinetic precision, and interval censoring may have led to minor misclassification. Modeling of antibody decay using log_2_-transformed titers approximates a continuous outcome but remains constrained by the ordinal nature of MAT data and the small number of seroconversions (*n* = 22), which reduces power and may explain the modest model fit (*R*^2^). These analyses should therefore be interpreted as indicative trends rather than exact quantitative estimates. The 18-week follow-up does not capture long-term decay, and the study’s focus on pregnant women from a single rural district further limits extrapolation.

## CONCLUSION

This study demonstrates frequent, asymptomatic *Leptospira* exposure and suggests rapid antibody decay among pregnant women in an endemic setting. These findings, in line with global cohort evidence, highlight a high level of silent transmission of leptospirosis, limitations of current surveillance tools, and reinforce the need for more sensitive, immunologically informed approaches. To address the hidden burden of leptospirosis, especially in underprofiled populations, public health systems must adopt improved diagnostic tools, inclusive risk assessments, and decay-aware epidemiological frameworks.
